# Comparative Analysis Between Laparoscopic Extravesical Repair and Laparoscopic O’Connor Repair for Supratrigonal Vesicovaginal Fistula

**DOI:** 10.5152/tud.2024.23147

**Published:** 2024-01-01

**Authors:** Yash Manharlal Tilala, Sabyasachi Panda, Amiya Shankar Paul, Pramod Kumar Mohanty, Sanjay Choudhuri, Samir Swain

**Affiliations:** Department of Urology and Renal Transplant, S.C.B Medical College and Hospital, Odisha, India

**Keywords:** Laparoscopic extravesical, laparoscopic O’Connor, laparoscopic vesicovaginal fistula repair, supratrigonal vesicovaginal fistula

## Abstract

**Objective:**

The objective of the study was to conduct a comparative analysis of various intraoperative parameters and postoperative outcomes between the laparoscopic extravesical repair versus the laparoscopic O’Connor repair techniques in management of supratrigonal vesicovaginal fistula.

**Methods:**

A prospective nonrandomized study was conducted from January 2018 to January 2023, in which 36 patients who met inclusion criteria like primary or recurrent, single, simple, supratrigonal vesicovaginal fistula were included. Among these patients 18 patients were operated with laparoscopic O’Connor repair, while 18 were operated with laparoscopic transperitoneal extravesical vesicovaginal fistula repair. Intraoperative and postoperative parameters of these 2 techniques were compared.

**Results:**

Laparoscopic O’Connor repair had longer operative time of 140 minutes, while laparoscopic extravesical VVF repair had an operative time of 117 minutes (*P* = .026). Mean blood loss was also significantly higher in laparoscopic O’Connor (210 mL versus 95 mL) (*P* = .004). Postoperative complications and analgesics requirement were less with laparoscopic extravesical repair. Hence, laparoscopic extravesical repair reduced mean hospital stay (3.2 days versus 3.9 days) (*P* = .003). A success rate of 83.33% for laparoscopic O’Connor and 94.45% for laparoscopic extravesical repair (*P* = .153) was recorded.

**Conclusion:**

Laparoscopic extravesical approach appears to be a convenient and effective method in selective supratrigonal vesicovaginal fistula repair.

## Introduction

**Main Points**

Laparoscopic extravesical approach is meticulous site specific dissection to expose the fistulous tract without cystotomy with or without vaginal closer.
Laparoscopic extravesical techniques is associated with relatively less operative time, less blood loss, less intravenous analgesic required, less post-operative complications, reduced duration of hospitalization.
Inadvertent cystotomy with difficult lap. extravesical repair can be salvaged with intra-operative adoption of lap. O’Connor technique.
Laparoscopic extravesical approach appears to be a convenient and effective method in selective supratrigonal VVF repair

The inadvertent urinary tract injury during pelvic surgery and complicated obstructed labor may cause the urogenital fistula (UGF). The most common UGF is vesicovaginal fistula (VVF). The reported rate of bladder injuries ranges from 0.1% to 0.49% and from 0.01% to 0.24% for the ureter.^1^ During surgery, mostly with an incidence of 1/1800 abdominal hysterectomies, inadvertent injuries occur due to electrocoagulation; hence, 70% of these injuries are diagnosed in the late postoperative period.^2^

The management of VVF repair either via transvaginal or transabdominal route depends on various factors but most importantly on surgeon’s own skills, experience, and preference. For supratrigonal VVF as well as whenever there is the need of ureteric reimplantation, bladder augmentation, or failed previous vaginal repair, the transabdominal approach is the most suitable option ranging from open, laparoscopic or robot-assisted methods of repair. However, the abdominal route has higher morbidity.^3^

Vesicovaginal fistula can affect the social, physical, mental, and sexual health of the patient due to continuous involuntary urine leakage and the need of undergoing another surgery for its management, which can be very stressful, especially if laparotomy is required.

Laparoscopic approach has gained much popularity in the management of supratrigonal VVF. It offers less morbidity and quicker recovery with better cosmetic outcomes. Laparoscopic O’Connor and laparoscopic transperitoneal extravesical VVF repair are the 2 main techniques widely performed for supratrigonal VVF repair with durable outcomes, but no data are available on the comparison between them. Hence, we conducted this prospective study to analyze the outcomes of laparoscopic O’Connor and laparoscopic extravesical techniques for supratrigonal VVF repair.

## Material and Methods

This prospective, nonrandomized, interventional, analytic, cohort study was conducted from January 2018 to January 2023.

Patients were included based on inclusion criteria such as patient with primary or recurrent, single, simple supratrigonal VVF. Patients with infratrigonal, multiple, complex, postradiation, malignant fistulas, urethrovaginal and ureterovaginal fistulas were excluded.

Ethical clearance was obtained as per norms from the institutional ethical committee of S.C.B Medical College and Hospital after full review (Approval No: 1272, Date: May 8, 2023).

The study population comprised 36 patients with supratrigonal VVF who met the inclusion criteria.

Valid, informed, and written consent was obtained from all the patients.

Statistical Analysis

Qualitative variables were analyzed with chi-square test or Fisher’s exact test as appropriate. Quantitative variables were compared with Student’s test or Mann–Whitney *U*-test as appropriate. *P* value < .05 was considered statistically significant. Statistical analysis was carried out with Statistical Package for the Social Sciences (SPSS) version 29.0 (IBM SPSS Corp.; Armonk, NY, USA).

### Preoperative Evaluation

All patients were evaluated with detailed history, physical examination, urethrocystoscopy, and vaginoscopy. There was a history of involuntary continuous urinary leakage per vagina in all patients. All patients were investigated with urine routine, microscopy and culture (sensitivity), renal function test, abdominal ultrasound, CT urography (to rule out ureterovaginal fistula), urethrocystoscopy, and vaginoscopy to assess the site, size, number of the fistula, distance to ureteric orifices or bladder neck. All patients had received clindamycin intravaginal suppository for 3 days prior to surgery.

Among 36 patients 18 patients were operated with laparoscopic O’Connor VVF repair whereas rest 18 patients were operated with laparoscopic transperitoneal extravesical VVF repair. In reconstructive surgery the first attempt, surgeon’s experience and his preference also play important factor in outcomes. So in this study the patients were selected for operative intervention by operating surgeon based on intraoperative urethrocystoscopy, vaginoscopy findings and his experience. All the patients have been operated by 2 surgeons who were well-versed and well experienced more than 7 years with both the techniques. In difficult laparoscopic extravesical repair, where extensive perivesical adhesions, long curved fistula tract, option of intraoperative adoption of laparoscopic O’Connor approach had been kept open by the surgeons.

## Surgical techniques

### Laparoscopic O’Connor VVF Repair

**Figure 1.**
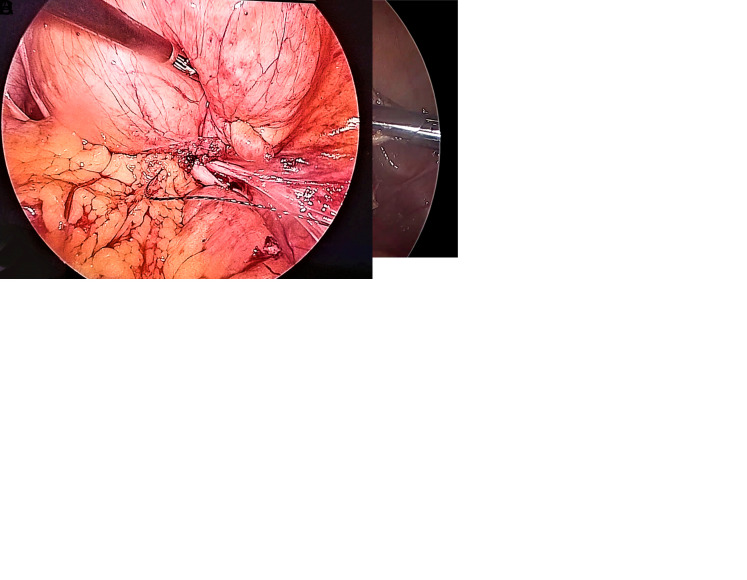
Laparoscopic O’Connor repair. (A) Ureteric catheter placed. (B) A plane between vagina and bladder has been created. (C) The fistulous tract was excised. (D) Saline leak test was performed to ensure watertight closure.

Under general anesthesia, in low lithotomy position, urethrocystoscopy and vaginoscopy was carried out and bilateral ureteric catheters were placed. Also another ureteric catheter was placed through the fistulous tract from the bladder and brought out from the vagina for easy identification of fistula (Figure 1A). An 18 Fr Foley catheter was placed and secured to both ureteric catheters. Sterile lignocaine jelly-soaked gauze pack was placed in the vagina. Pneumoperitoneum was achieved using a Veress needle and 10 mm supraumbilical port was placed. The patient was then tilted to a 15-30° Trendelenburg position. Two working ports, 10 mm at the right iliac fossa and 5 mm at the left iliac fossa over the spinoumbilical line, were placed. Another 5 mm trocar was placed in the lower abdomen as per requirement.

After adhesiolysis the plane was created between bladder and vagina by identification via gentle push of vaginal pack and movement of ureteric catheter placed in the fistulous tract (Figure 1B). A limited vertical cystotomy was performed. Then the fistula was identified, cystotomy was then extended 1-1.5 cm all around the fistula and excision of the fistula (Figure 1C). In single layer vaginal opening was repaired with polyglactin/V-Loc 3.0 ½ circle needle in a continuous manner placing the suture line horizontally.

Cystotomy was closed in a continuous manner in a vertical orientation to get a nonoverlapping suture line with respect to the vaginal suture line with a 2-0 polyglactin/V-Loc 3.0 ½ circle needle. The repair was augmented with either greater omentum or epiploic appendix of sigmoid colon as per availability. Then the bladder was filled with about 200-300 mL of saline to ensure a watertight repair (Figure 1D). We did not encounter any leak. An 18 Fr Ryle’s tube was used as a pelvic drain.

No suprapubic catheter (SPC) was placed. The 10 mm trocar sites were closed with 1-0 polyglactin.

### Laparoscopic Transperitoneal Extravesical Vesicovaginal Fistula Repair

**Figure 2.**
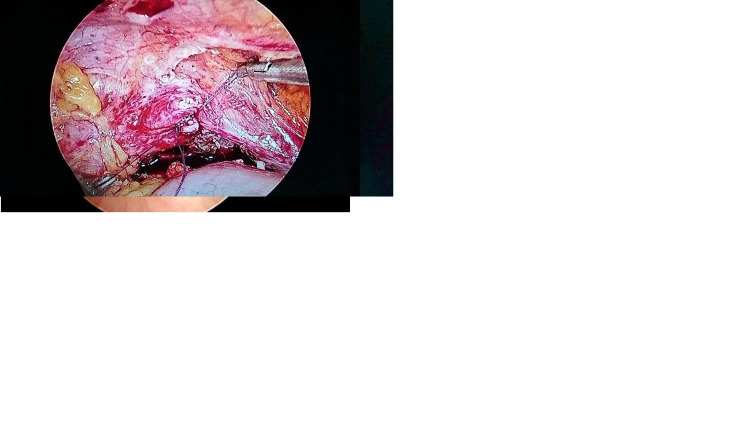
(A) Laparoscopic extravesical repair cystoscopic examination to confirm location. (B) Site-specific dissection of fistula. (C) Single-layer closure of the fistula tract.

Under general anesthesia in low lithotomy position cystoscopy (Figure 2A) and vaginoscopy was performed. Placement of bilateral ureteric catheter, another ureteric catheter through the fistula, placement of Foley’s catheter, sterilized lignocaine jelly-soaked vaginal pack, and port placement was the same as described in the laparoscopic O’Connor technique.

The urinary bladder was mildly filled with normal saline. Vaginal pack was gently pushed in to identify the plane between the urinary bladder and vaginal vault and sometimes we used to give downward gentle pressure with vaginal pack this help us to deflect the vagina downward, for more precise delineation of plane and it provides traction during dissection. The already placed ureteric catheter in the VVF tract was moved with back-and-forth movement to delineate the exact position of the fistula tract in bladder and then meticulous site specific dissection was done to expose the fistulous tract without cystotomy. The ureteric catheter was then brought out from the fistula tract (Figure 2B). We neither trimmed nor excised the fistula tract.

After adequate dissection a single-layered closure of the fistulous tract was performed with 2-0 Vicryl/V Loc 3.0½ circle needle (Figure 2C). The vaginal vault was not closed most of the time. The greater omentum or appendix epiploica, as per availability, has been used as the interposition flap in all the cases. With 200-300 mL of saline the bladder was filled to check any leak. We did not encounter any leak. We did not place an SPC. An 18-Fr Ryle’s tube placed as pelvis drain. The 10 mm ports were closed with 1.0 polyglactin.

### Postoperative Management and Follow-Up

Oral liquids along with anticholinergic medication were allowed as per patient’s tolerance. On first postoperative day, the vaginal pack was removed. Ureteric catheters were removed 48-96 hours after surgery depending upon the intraoperative findings. The drain was removed once the output was below 50 mL/day after the removal of ureteric catheter. Patients were discharged on per-urethral catheter when tolerating orally, complete ambulatory and on adequate analgesic as well as anticholinergic until the removal of the per-urethral catheter. On the 14th postoperative day micturating cystourethrogram (MCU) was done in all patients and if there was no leak then the per-urethral catheter was removed. Patients were advised to avoid sexual intercourse for 3 months. All patients were followed up postoperatively strictly for the first visit at 3 months, then 6, 9, and 12 months in the first year and every 6 months thereafter.

In follow-up, all the patients have been evaluated with a detailed history and physical examination which emphasis particularly on detecting urinary leakage. If patients had no such history, symptoms or signs of urinary leakage they were considered as successful repair.

## Results

Baseline characteristics were comparable between both groups (Table 1). Mean age was 45.2 years in the laparoscopic O’Connor group, while it was 39.8 years in the laparoscopic extravesical group. BMI was almost comparable in both groups. There were 33.3% patients in laparoscopic O’ Connor group and 27.7% patients in laparoscopic extravesical group had comorbidities. As the obstetric care has improved, the most common etiology observed for VVF was gynecological, in the form of abdominal hysterectomy of 61.1% in the laparoscopic O’Connor group and 72.2% in the laparoscopic extravesical group. The most common type of hysterectomy observed was Wertheim hysterectomy. Mean duration of symptoms in both the groups suggests delayed repair; it is mainly due to late presentation of the patients to us. The mean size of fistula was 19.6 mm in the laparoscopic O’Connor group and 12.8 mm in the laparoscopic extravesical group. Both the groups had equal primary and recurrent VVF cases.

Intraoperative and postoperative data analysis showed that (Table 1) the mean operative duration was 117 minutes in the laparoscopic extravesical group, which was significantly less in comparison to the laparoscopic O’Connor group (140 minutes) (*P* = .026). All the patients have been repaired with interposition of greater omental flap or appendix epiploica as per availability. Mean blood loss was also significantly less in the laparoscopic extravesical group (95 mL), whereas in the laparoscopic O’Connor group it was 210 mL (*P* = .004), and no one had a blood transfusion. Mean postoperative requirement of IV analgesics (tramadol) was significantly low in the laparoscopic extravesical group (180 mg) and 250 mg in the laparoscopic O’Connor group (*P* = .024). Most common postoperative complication was hematuria in both the groups. Overall postoperative complications were higher side in the laparoscopic O’Connor group (44.35%) in comparison to laparoscopic extravesical group (16.6%). Post operatively early convalescence and significantly less duration of hospitalization were observed in the laparoscopic extravesical group (mean 3.2 days), whereas in the laparoscopic O’Connor group the duration of hospitalization was 3.9 days (*P* = .003). Mean duration of ureteric catheter removal was almost comparable in both the groups. Mean follow up duration in laparoscopic O’Connor group was 12.4 months, versus 14.71 months in laparoscopic extravesical group (*P* = .657), with recorded success rate of 83.33% in laparoscopic O’Connor group versus 94.45% in laparoscopic etravesical group (*P* = .153). There was no laparoscopic to open conversion. Five patients (27.7%) in the laparoscopic O’Connor group had urge urinary incontinence (UUI), which was successfully managed with an anticholinergic for 4 weeks, whereas no such complaints were noted in the laparoscopic extravesical group. Three patients (16.67%) from the laparoscopic O’Connor group and 1 patient (5.5%) from the laparoscopic extravesical group had vaginal leak in the postoperative MCU study and were put on per-urethral catheter with an anticholinergic for 4 weeks but they failed to respond; later, at 12 weeks they again underwent laparoscopic O’Connor repair and laparoscopic extravesical repair, respectively, with successful outcomes. These patients were not included in this study.

## Discussion

Management of VVF is directed toward the immediate symptomatic relief from involuntary urinary leakage and restoration of the urogenital functions.

Among the 2 approaches of VVF repair, vaginal and transabdominal approaches, the vaginal approach is usually preferred for infratrigonal VVF, while for supratrigonal VVF the transabdominal approach is considered the preferred one given the advantage of its reproducibility and familiarity. For transabdominal supratrigonal VVF repair, the O’Connor technique has been considered as the gold standard, with reported success rates of >85%.^4,5^ But it is associated with morbidities and longer convalescence period.

The current paradigm of management of supratrigonal VVF repair has shifted rapidly toward the laparoscopic approach, as it fulfills all the basic principles of VVF repair with its well-known benefits along with its efficacy, feasibility, safety, reproducibility and good successful outcomes achieved with less morbidity, early convalescence, and better cosmetic results.^6-8^

The first laparoscopic VVF repair was reported by Nezhat and associates in a single patient^9^ and then in 1994 they reported in a series of bladder repair in 19 patients.^10^ In 1998, von Theobold described the laparoscopic extravesical VVF repair, which was the site-specific dissection of the bladder away from the vagina and a single-layer bladder closure, as “closure of the vagina was not necessary.”^11^ A few months later, Miklos et al^12^ described a laparoscopic extravesical technique utilizing a 3-layer closure. Thereafter many case reports and case series on laparoscopic O’Connor and extravesical VVF repair techniques have been published (Tables 2 and 3), but there had been a lack of comparison between these 2 techniques. Thus, the present study was conducted. As most of the studies have shown that the hysterectomy as the most common cause of VVF. Post-hysterectomy fistulas are generally supratrigonal in location and usually are associated with minimal inflammation and can be repaired early without compromising the outcomes.^13^ The laparoscopic extravesical group in comparison with laparoscopic O’Connor group has shown less operative duration, less blood loss, and reduced duration of hospitalization.

The mean operative duration ranged from 85 to 380 minutes in various laparoscopic O’Connor repair studies, while in our study the mean duration of laparoscopic O’Connor was 140 minutes and it is comparable with Sumit Sharma et al’s^15^ study, in which a series of 22 laparoscopic VVF repair were carried out. In laparoscopic extravesical repair the mean operative duration was significantly reduced to 117 minutes in comparison to the laparoscopic O’Connor group. We preferred to use the barbed sutures most of the time in both techniques, as it provides more secure closure, obviating the need of knotting.^16^ As in extravesical repair it was a site specific dissection without excision of fistula and without cystotomy the mean operative duration was less. Additionally, not closing the vaginal vault also contributes in time saving without compromising the surgical outcomes.^17^ As we did not close the vaginal vault in most of the patients undergoing the laparoscopic extravesical technique, we did not notice any complication in the postoperative period and postoperative vaginoscopy was indifferent compared to the patients in whom the vaginal vault was closed.

With comparable mean duration of bilateral ureteric catheter removal, 2.9 days in laparoscopic O’Connor whereas 2.5 days in laparoscopic extravesical, none of the patients had any leak per vagina. The mean dosage of IV analgesics (tramadol) in the laparoscopic extravesical group was significantly lower in comparison to the laparoscopic O’Connor group.

Lesser operative time, comparatively early removal of bilateral ureteric catheter and less analgesic required ultimately results in early convalescence and reduced duration of hospitalization, 3.2 days in laparoscopic extravesical versus 3.9 days in laparoscopic O’Connor, which is comparable with published literature. Regarding the blood loss, the mean blood loss in the laparoscopic O’Connor group was 210 mL, whereas in published literature of laparoscopic O’Connor it ranged from 59 to 333 mL. On the other hand, in the present study, the mean blood loss was 95 mL with laparoscopic extravesical technique, while with the case series of laparoscopic extravesical repair the Abdel Karim et al^27^ study reported a mean blood loss of 110 mL and duration of hospitalization was 3 days, which is comparable with ours, 3.2 days of hospitalization. The reduced mean blood loss in laparoscopic extrvesical was mainly due to limited dissection and precluded cystotomy that could be a source of bleeding. In the present study, we observed that in the patients with a long curved supratrigonal fistulous tract the risk of inadvertent cystotomy during the laparoscopic extravesical technique was slightly on the higher side. In such cases if inadvertent cystotomy occurred then adoption of laparoscopic O’Connor approach as salvage procedure intraoperatively was we found as a good option. We had 1 such case that has been included in the laparoscopic O’Connor group. Perivesical adhesions can also cause inadvertent cystotomy during laparoscopic extravesical repair; however, we had no such case of conversion due to perivesical adhesions. In cases where the fistulous opening was in close proximity of the ureteric orifice laparoscopic O’Connor has advantageous cystotomy as it can help in identification of the fistula tract and precise excision of the tract with securing bilateral ureteric orifice.

In the post-operative period, 27.7% patients had Clavien–Dindo class I complications in the laparoscopic O’Connor group, like mild hematuria, which was resolved spontaneously in 1-2 days, also prolonged paralytic ileus in 11.1% and surgical site infection (SSI) in 5.55% of the patients, which were managed successfully with conservative measures. On the other hand, in the laparoscopic extravesical group, these complications were very limited, with 11.1% of the patients having hematuria and 5.55% having prolonged paralytic ileus, which were managed conservatively. With comparable mean durations of follow-up, 12.4 months in the laparoscopic O’Connor group and 14.7 months in the laparoscopic extravesical group, 26% of the patients in the laparoscopic O’Connor group had UUI. It was probably due to bladder spasms after cystotomy during laparoscopic O’Connor repair. However, these patients were managed with anticholinergics. In the present study the success rate of laparoscopic O’Connor was 83.33%, while in literature it ranges between 87% and 100%. We would like to quote that 3 failed cases in laparoscopic O’Connor and 1 in laparoscopic extravesical have been reoperated at an interval of 12 weeks with laparoscopic O’Connor and laparoscopic extravesical techniques, respectively, with successful outcomes. It denotes the reproducibility of these techniques. The limitations of this study are the small sample size and nonrandomization of the patients. However, size of VVF, comorbidities of the patients, and primary or recurrent cases were comparable and without significant differences in both the groups.

In conclusion, we observed that laparoscopic extravesical technique is more patient friendly with lesser operative time, less blood loss, reduced duration of hospitalization, and successful outcomes in comparison to laparoscopic O’Connor technique in the management of supratrigonal VVF. However, multi-institutional study outcomes should be sought.

## Figures and Tables

**Table 1.** Baseline Characteristics of the Patients and Surgical Outcome

Variables	Laparoscopic O’ Connor VVF Repair (n = 18)	Laparoscopic Extravesical VVF Repair (n = 18)	*P*
Age (years)	45.2 (28-61)	39.8 (28-58)	.116
BMI (kg/m^2^)	21.7 (20-25)	20.7 (18-23)	.067
Comorbidities (n, %)	HTN: 2 (11.1%) DM II + HTN: 4 (22.2%)	HTN: 2 (11.1%) DM II: 2 (11.1%) Hypothyroidism: 1 (5.55%)	.678
Etiology (n, %)	TAH + BSO: 8 (44.4%) TAH: 3 (16.7%) NDVH: 4 (22.2%) LSCS: 1 (5.55%) Obstructed labor: 2 (11.1%)	TAH + BSO: 9 (50%) TAH: 4 (22.2%) NDVH: 3 (16.7%) Obstructed labor: 2 (11.1%)	.201
Duration of symptoms (months)	5.06 months (2-12)	6.2 months (3-12)	.382
Size of VVF (mm)	19.6 mm (15-25)	12.8 mm (10-18)	.232
Primary/recurrent fistulas (n, %)	Recurrent VVF: 3 (16.7%) Primary: 15 (83.3%)	Recurrent VVF: 3 (16.7%) Primary: 15 (83.3%)	3
Operative duration (minutes)	140 (95-210)	117 (90-140)	.026
Blood loss (ml)	210 mL (80-300)	95 mL (70-150)	.004
IV analgesics usage (tramadol) (mg)	250 mg (100-400)	180 mg (100-300)	.024
Period of hospitalization (days)	3.9 days (3-5)	3.2 days (3-4)	.003
Removal of ureteric catheter (days)	2.9 (2-4)	2.5 (2-3)	.086
Postoperative complications (n) (%)	Hematuria: 5 (27.7%) Prolonged paralytic ileus: 2 (11.1%) SSI: 1 (5.55%)	Hematuria: 2 (11.1%) Prolonged paralytic ileus: 1 (5.55%)	.047
Follow-up (months)	12.4 (3-36)	14.7 (3-48)	.657
Complication during follow-up (n, %)	UUI: 5 (27.7%)	0%	.032
Success rate %	83.33% (15/18)	94.45 % (17/18)	.153
Recurrence rate %	16.67% (3/18)	5.55 % (1/18)	.071
3	No laparoscopic to open conversion		3

DM II, diabetes mellitus type II; HTN: Hypertension; LSCS, lower segment caesarean section; NDVH, nondescent vaginal hysterectomy; TAH + BSO, total abdominal hysterectomy with bilateral salpingo-oophorectomy; SSI, surgical site infection, TAH, total abdominal hysterectomy; UUI, urge urinary incontinence.

**Table 2.** Various Studies of Laparoscopic O’Connor Vesicovaginal Fistula Repair

Study	Number of Patients	Mean Age (Years)	Etiology (m.c.)	Mean Op. Time (Minutes)	Mean Blood Loss (mL)	Mean Hospital Stay (Days)	Foley Removal Time (Days)	Follow-Up (Months)	Success Rate (%)
Present study (2023)	18	45.2	Hysterectomy	140	210	3.9	14	14.7	83.33
Bastab Gosh et al^14^ (2016)	13	36	Hysterectomy	153	59	4	11	18	100
Sumit Sharma et al^15^ (2014)	22	38	Hysterectomy	140	75	6	NR	3.2	N.R
Ali Serdar Go¨ zen et al^18^ (2007)	3	41	Hysterectomy	163	333	6	10	19	100
Das Mahapatra et al^19^ (2007)	12	34	Hysterectomy (7) + Obs (5)	166	125	5.5	14	3-36	91.7
Nagraj et al^20^ (2007)	13	N.R.	Hysterectomy	130	N.R	4.5	15	21	91.6
Wong et al^21^ (2006)	2	N.R.	Hysterectomy	380	<100	3	21	40	N.R.
Sotelo et al^22^ (2005)	15	38	Hysterectomy (14) + Obs (1)	170	N.R.	3	10	26	93.3
Chibber et al^23^ (2005)	8	N.R.	Hysterectomy (6) + Obs (2)	220	NR	3	14	3-40	87.5
Ou Cs et al^24^ (2004)	2	N.R.	Hysterectomy	NR	NR	NR	14-20	3	N.R
Hemal et al^25^ (2001)	2	N.R.	N.R.	140	100	3	21	6	100
Nezhat et al^9^ (1996)	19	N.R.	N.R.	N.R.	N.R.	N.R.	7-14	6-48	95
Nezhat et al^10^ (1994)	1	N.R.	Hysterectomy	85	100	1	14	10	100

Op., operative; N.R., no record; Obs., obstetrics; m.c., most common.

**Table 3.** Various studies of Laparoscopic Extravesical Vesicovaginal Fistula Repair

Study	Number of Patients	Mean Age (Years)	Etiology (m.c.)	Mean Op. Time (Minutes)	Mean Blood Loss (mL)	Mean Hospital Stay (Days)	Foley Removal Time (Days)	Follow-Up (Months)	Success Rate (%)
Present study (2023)	18	39.8	Hysterectomy	117	95	3.2	14	14.7	94.5.%
Miklos,et al^26^ (2014)	43	46.5	Hysterectomy (95%)	144.8	51	1.2	14-21	17.3	98%
Abdel-Karim et al^27^ (2011)	15	N.R.	Hysterectomy	172	110	3	21	19	100%
Lee et al^28^ (2010)	5	N.R.	Hysterectomy	95	3	5	14	56	100 %
Tiong et al^29^ (2007)	1	44	Hysterectomy	260	<100	1	21	6	100%
Miklos et al^12^ (1999)	1	N.R.	N.R.	N.R.	N.R.	N.R.	21	6	0%
Theobald et al^11^ (1988)	1	N.R.	Hysterectomy	70	100	8	7	6	100%

Op., operative; N.R., no record; m.c., most common.
